# Characteristics of Lower Limb Dominant and Nondominant Joint Load Changes After Long-Distance Running in Young Male Runners Under OpenSim Environment

**DOI:** 10.3390/s25206301

**Published:** 2025-10-11

**Authors:** Xiaocan Li, Lijuan Mao

**Affiliations:** School of Athletic Performance, Shanghai University of Sport, Shanghai 200438, China; 2411811013@sus.edu.cn

**Keywords:** OpenSim, joint contact force, dominant side, one-dimensional statistical parameter mapping

## Abstract

This study aims to investigate the characteristics of load changes in the hip, knee, and ankle joints of the dominant and non-dominant lower limbs of young male runners after long-distance running. Using the OpenSim public dataset (containing bilateral biomechanical data before and after long-distance running from 20 young male runners), personalized musculoskeletal models were established. Contact forces in three directions at lower limb joints during the running stance phase were calculated. Statistical analysis employed one-dimensional statistical parameter mapping (SPM1d) and two-factor repeated measures ANOVA (time × side). Results revealed significant time × side interaction effects (*p* < 0.05) for contact forces in the medial–lateral direction at the hip, the anterior–posterior direction at the knee, and all three directions at the ankle. Simple effects analysis showed that post-run medial–lateral hip forces significantly increased during the push-off phase, while anterior–posterior ankle forces significantly increased during the mid-to-late stance phase on both sides (d = 0.718–1.002). For the superior–inferior direction at the hip and knee, only main effects of time or side were present. Post-run joint contact forces significantly increased, with the dominant side consistently exceeding the non-dominant side across multiple stance and push-off phases (d = 0.58–1.6), indicating stable side-to-side differences. These findings indicate that long-distance running not only increases multi-joint loading in the lower limbs but also exacerbates asymmetry between the dominant and non-dominant sides during the initial stance and push-off phases. This redistribution of load, coupled with bilateral control imbalance, may further elevate the risk of injury.

## 1. Introduction

With the increasing awareness of national fitness, running has become one of the most popular exercise programs due to its simplicity and benefits. Several studies have shown that running reduces all-cause mortality, cancer mortality, and cardiovascular disease mortality and helps to reduce body weight and improve cardiovascular and cardiopulmonary function at rest [1]. Despite the proven benefits of running, overuse injuries associated with running are common, with nearly 50% of runners sustaining musculoskeletal injuries from running each year [2].

The lower limbs have different functional distinctions bilaterally during running, with the dominant limb primarily generating energy and the nondominant limb responsible for maintaining balance and stability [3]. Differences in functional tasks may lead to some degree of asymmetry in the lower limbs. A growing body of evidence suggests that such biomechanical asymmetry is a significant risk factor for running-related injuries [4]. For instance, asymmetric loading has been specifically associated with a higher risk of stress fractures and overuse syndromes [5]. Motor control theory suggests that the presence of asymmetry represents a potential constraint that limits an athlete’s movement strategy [6]. This can lead athletes to adopt motor behaviors that increase the risk of injury. Potential mechanisms of injury include athletes performing tasks in an inefficient or dysfunctional manner, leading to fatigue or accumulation of microtrauma. Additionally physical limitations such as restricted joint function may lead athletes to adopt postures during exercise that are detrimental to the health of muscles or joints, thereby increasing the likelihood of injury [7]. Asymmetrical running gait patterns have been extensively studied and have been identified as a significant risk factor for overuse injuries in runners. During running the body is repeatedly subjected to high impact forces generated by the collision of the foot with the ground. The high impact forces to which the body is subjected and the changes in the attenuation of these forces when the runner is in a fatigued state are expected to lead to overuse-related injuries [8]. Studies have shown that increased asymmetry is associated with disturbances in lower extremity joint loading [9]. Altered or even cumulative biomechanical loading may lead to potential running-related injuries and thus affect running performance [10,11]. Therefore, it is particularly important to explore the changes in lower limb loading bilaterally after long-distance running.

However, direct in vivo measurement of joint contact forces (JCFs) is invasive [12]. While it enables highly precise assessment of tissue loading [13], the anatomical and physiological structures within the joint region are altered by the surgical procedure, and implementation in healthy runners is challenging [14]. OpenSim is an open-source software platform widely used in biomechanics for creating and analyzing dynamic simulations of movement [15]. It enables users to build complex musculoskeletal models by defining rigid bodies, joints, and muscle components. These models can be personalized by adjusting parameters like muscle geometry to suit specific research needs, allowing for the calculation of critical but otherwise unmeasurable in vivo data, such as muscle and joint contact forces during activity [16]. This capability provides a credible method for investigating the loading patterns that are key to understanding potential injury mechanisms. For data processing and analysis, this study used one-dimensional statistical parameter mapping (spm1d) for the analysis of each index; these indexes are usually described by one-dimensional kinematic trajectories and force trajectories. Due to the difficulty of analyzing these trajectories directly, many studies choose some scalars that can summarize the characteristics of the trajectories such as the mean and maximum values to perform statistical tests, which can simplify the analysis process but focuses on specific peaks and lacks holistic evaluation. In contrast, spm1d, as a method of biomechanical analysis, can evaluate the statistical significance of the entire motion profile [17]. For example, statistical parametric mapping (SPM) has been widely used in the field of neuroimaging to see changes throughout the brain rather than just in specific regions [18]. By using spm1d, researchers can gain a more comprehensive understanding of movement biomechanics rather than focusing only on the peaks of specific movements [19].

In summary this study aims to investigate the load change characteristics of the dominant and non-dominant sides of the lower limbs of young male runners after long-distance running through opensim musculoskeletal simulation, so as to clarify the load changes in the various stages of the support period in order to target personalized intervention on both sides and to provide a theoretical basis for the science of exercise in the running population, so as to reduce the occurrence of injuries. The research hypothesis of this study is that the contact force of the lower limb joints will increase significantly after long-distance running, and bilateral asymmetry will be exacerbated at the early stage of support in the running support period.

## 2. Materials and Methods

### 2.1. Subjects

The experimental subjects for this paper were taken from a publicly available dataset [16] available at https://doi.org/10.1016/j.heliyon.2022.e11517, accessed on 18 June 2025, which contained 20 male recreational runners (age: 25.8 ± 1.6 years, weight: 67.8 ± 5.3 kg, height: 1.73 ± 0.05 m). All participants were right-leg dominant, which meant they preferred the right leg for kicking. All participants had a history of terrestrial or treadmill running activity (5–8 km per session, 3–6 times per week) and did not have any lower limb injuries or foot deformities in the six months prior to testing.

### 2.2. Experimental Protocol

Subjects first underwent a 10 min walking or running warm-up on a treadmill or on the ground, followed by a 5 km pre-run test consisting of three running tests, one while static, and another two on the right and left foot. Kinematic and kinetic data were recorded simultaneously at 200 Hz and 1000 Hz with a Vicon motion capture system and AMTI dynamometer, respectively. The 5 km run was performed at 12 km/h (80% of the personal optimal speed), and the pre-run test was repeated immediately after the run. Three tests were conducted on each side. This simulation employed a full-body musculoskeletal model based on OpenSim, which has been thoroughly validated and widely adopted in gait and motion simulation research [20], providing a reliable analytical foundation for this study. To more accurately simulate the unique kinematic characteristics of long-distance running, several improvements were implemented based on established methodologies in the literature: the knee joint incorporates two additional degrees of freedom for abduction–adduction and internal–external rotation beyond the original sagittal plane flexion–extension, following a previously validated approach [21]. Simultaneously, muscle pathways were added to the cylindrical envelope surface to accommodate the previously used elliptical envelope surface [22]. Additionally, the extra range of motion in the coronal and horizontal planes of the knee joint was updated. The ankle joint was modeled as a pivot joint, permitting dorsiflexion–plantarflexion. During data processing, the tarsometatarsal and metatarsophalangeal joints were locked to simplify the running task model [21]. This study defines the stance phase as the period from heel contact to toe-off. Heel contact and toe-off events were identified as the time points when the vertical ground reaction force exceeded and fell below a threshold of 20 N, respectively. The stance phase was subsequently subdivided into the initial stance phase (0–20%), mid-stance phase (20–50%), and push-off phase (50–100%) [23].

### 2.3. Joint Contact Force Calculation

**Figure 1:**
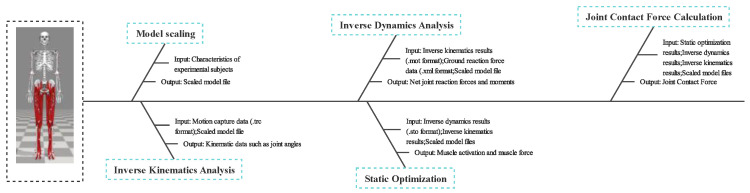
Joint contact force calculation process.

The main indexes in this study are bilateral lower limb contact forces in three directions for the hip, knee and ankle joints, and the calculation steps are as follows: Firstly, the model is scaled according to the characteristics of the experimental subjects, and the static marker point data and model files are input; inverse kinematic analysis is conducted on the motion capture data (.trc format) and scaled model files to obtain the joint angles and other kinematics data. Inverse dynamics analysis requires the input of inverse kinematics results (.mot format), ground reaction force data (.xml format), and scaled model files to compute net joint reaction forces and moments; static optimization requires the input of inverse kinetics (.sto format), inverse kinematics results, and scaled model files to determine muscle activation and muscle force; and finally, we used the static optimization, inverse dynamics, inverse kinematics results, and scaled model files to obtain the desired joint contact forces, Figure 1.

### 2.4. Statistical Analysis

The data was analyzed using a two-way repeated measures ANOVA combined with a one-dimensional statistical parametric mapping (SPM) approach, which included two within-group factors, time (pre-run, post-run) and side (dominant side, non-dominant side), with the dependent variable being joint contact force data from a continuous time series of gait cycles. The spm1d.stats.anova2rm function was used to generate time-series F-statistics to assess the following effects: Main effect of time: overall difference between pre- and post-running; main effect of side: inherent difference between dominant and nondominant sides; and time × side interaction effect. Interaction effect: whether running significantly changed the difference between the two sides. The significance threshold was set at α = 0.05, and multiple comparisons were corrected using random field theory (RFT). If the interaction effect was significant, a post hoc paired t-test was further conducted using the spm1d.stats.test_paired function to compare the change in the two-sided difference (dominant side vs. non-dominant side) between pre- and post-running, and to locate the time period in which the difference was significant; if the main effect was significant but the interaction effect was not, the overall effect of time or side was examined separately, and the effect quantities were reported by η^2^ (main effect) and Cohen’s d (pairwise comparisons). All kinematic and kinetic trials were visually inspected for quality, and no data gaps or obvious outliers were present; therefore, all successful trials from all subjects were included in the final analysis.

#### One-Dimensional Statistical Parameter Mapping (SPM1d) Analysis

SPM1d evaluates statistical differences across the entire time series (gait support phase) by constructing a continuous F-statistic curve, rather than at specific time points. When the F-curve exceeds the significance threshold (α = 0.05) corrected based on random field theory (RFT), the corresponding time interval (referred to as a ‘suprathreshold cluster’) is considered to exhibit a significant statistical effect.

## 3. Results

### 3.1. Bilateral Ankle Contact Force Before and After Running

**Figure 2:**
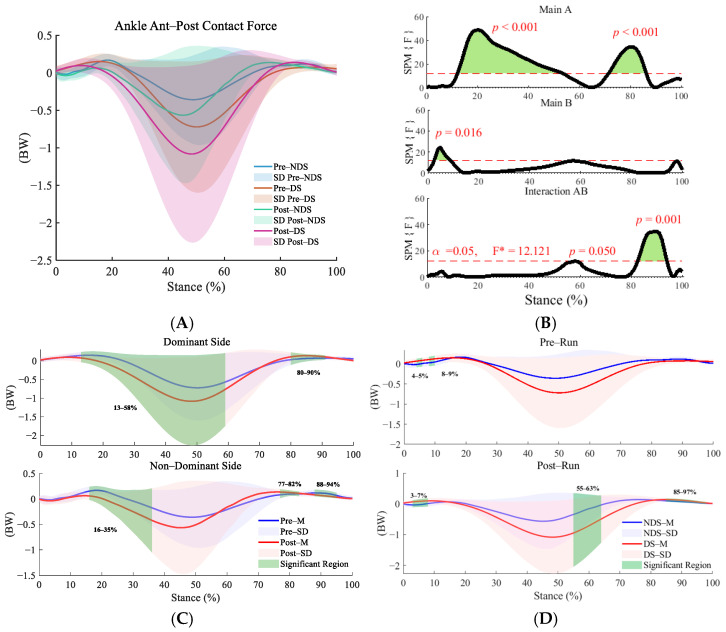
Analysis of anterior–posterior contact forces at the ankle joint. (**A**): Mean ± SD curves for dominant side (DS) and non-dominant side (NDS) before and after running; (**B**): analysis of main effects of time and side, and their interaction. Black solid lines represent SPM [F] statistics, red dashed lines denote significance thresholds, and green shaded areas indicate regions of statistical significance; (**C**): simple effects analysis for dominant and non-dominant sides; (**D**): simple effects analysis before and after running. SPM[F]: Represents a statistical parameter map constructed using the F-statistic within the statistical parametric mapping framework. This map displays the value of the F-statistic at each time point or spatial location, enabling a visual identification of regions exhibiting significant differences. F* is the F statistic calculated in analysis of variance. M denotes the mean, and SD denotes the standard deviation. The figure below is the same as above.

**Figure 3:**
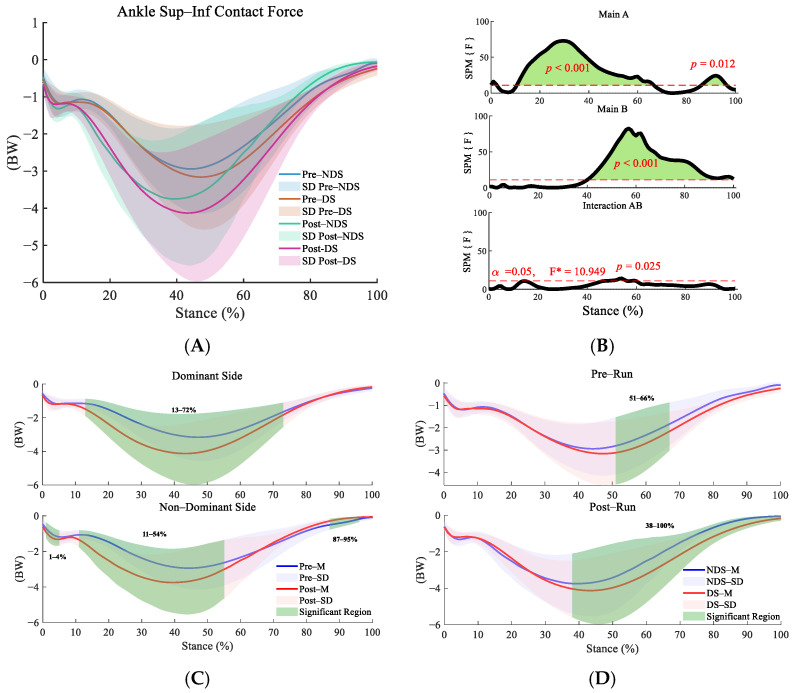
Analysis of superior–inferior contact forces at the ankle joint. (**A**): Mean ± SD curves for dominant and non-dominant sides before and after running; (**B**): analysis of main effects of time and side, and their interaction. Black solid lines represent SPM [F] statistics, red dashed lines denote significance thresholds, and green shaded areas indicate regions of statistical significance; (**C**): simple effects analysis for dominant and non-dominant sides; (**D**): simple effects analysis before and after running. M denotes the mean, and SD denotes the standard deviation. “SPM [F]” is clarified as “statistical parametric mapping F-statistic curve.” F* is the F statistic calculated in analysis of variance.

**Figure 4:**
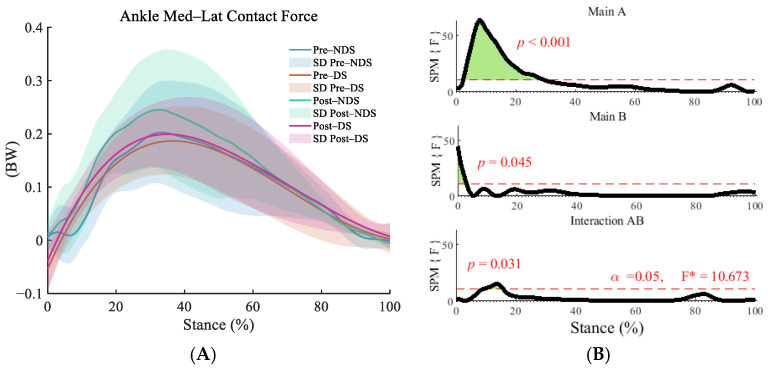

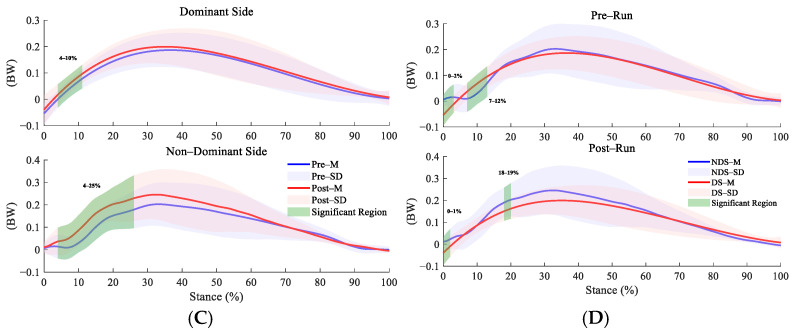
Analysis of medial–lateral contact forces at the ankle joint. (**A**): Mean ± SD curves for dominant and non-dominant sides before and after running; (**B**): analysis of main effects of time and side, and their interaction. Black solid lines represent SPM [F] statistics, red dashed lines denote significance thresholds, and green shaded areas indicate regions of statistical significance; (**C**): simple effects analysis for dominant and non-dominant sides; (**D**): simple effects analysis before and after running. M denotes the mean, and SD denotes the standard deviation. “SPM [F]” is clarified as “statistical parametric mapping F-statistic curve.” F* is the F statistic calculated in analysis of variance.

This study employed repeated measures ANOVA and found significant interaction effects between time and side across the anterior–posterior (F = 12.121, *p* = 0.001, η^2^ = 0.320; Figure 2B), superior–inferior (F = 10.949, *p* = 0.025,η^2^ = 0.230; Figure 3B),and medial–lateral directions (F = 10.949, *p* = 0.025, η^2^ = 0.144; Figure 4B). Post hoc analyses revealed significant increases in ankle contact force on both the dominant sides (DS) and non-dominant sides (NDS) during multiple phases of the support cycle (13–58% in the anterior–posterior direction) and the push-off phase (80–90% in the anterior–posterior direction, Figure 2C) following running (*p* < 0.01, d = 0.473–1.495). Lateral differences were already present before running (3–6% in the anterior–posterior direction), and these differences further widened after running, particularly during the push-off phase (55–63% and 85–97% in the anterior–posterior direction) and the mid-to-late stance phase (38–100% in the superior–inferior direction, Figure 3D).

### 3.2. Bilateral Knee Contact Force Before and After Running

**Figure 5:**
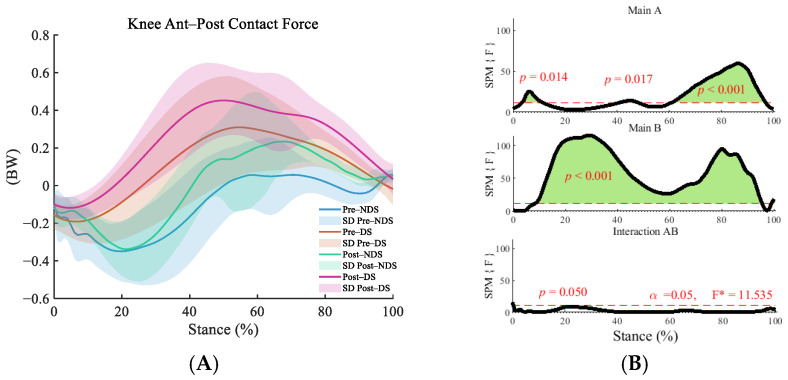

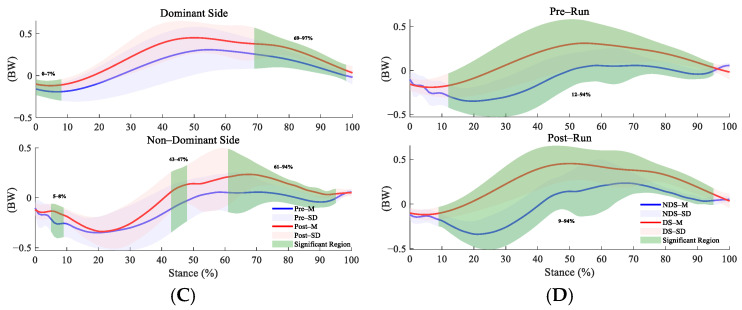
Analysis of anterior–posterior contact forces at the knee joint. (**A**): Mean ± SD curves for dominant and non-dominant sides before and after running; (**B**): analysis of main effects of time and side, and their interaction. Black solid lines represent SPM [F] statistics, red dashed lines denote significance thresholds, and green shaded areas indicate regions of statistical significance; (**C**): simple effects analysis for dominant and non-dominant sides; (**D**): simple effects analysis before and after running. M denotes the mean, and SD denotes the standard deviation. “SPM [F]” is clarified as “statistical parametric mapping F-statistic curve.” F* is the F statistic calculated in analysis of variance.

In the anterior–posterior direction, the interaction effect between time and side was significant at the moment of ground contact (1%) (F = 11.535, *p* = 0.05, η^2^ = 0.128) (Figure 5B). Post hoc tests revealed significantly increased ankle contact forces on both the dominant side and non-dominant side during the initial stance phase (dominant side: 1–7%; non-dominant side: 5–8%, 43–47%) and push-off phase (dominant side: 69–97%; non-dominant side: 61–94%) (*p* < 0.01) (Figure 5C). Pre-run differences existed between sides at 12–94% (*p* < 0.001), while post-run differences further expanded to 9–94% (*p* < 0.001) (Figure 5D).

**Figure 6:**
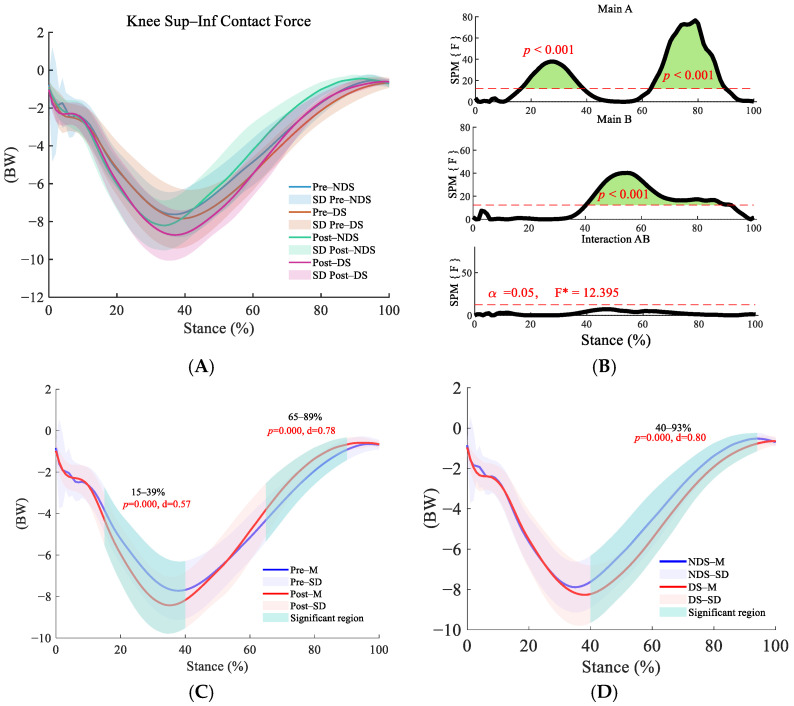
Analysis of superior–inferior contact forces at the knee joint. (**A**): Mean ± SD curves for dominant and non-dominant sides before and after running; (**B**): analysis of main effects of time and side, and their interaction. Black solid lines represent SPM [F] statistics, red dashed lines denote significance thresholds, and green shaded areas indicate regions of statistical significance; (**C**): simple effects analysis before and after running; (**D**): simple effects analysis for dominant and non-dominant sides. F* is the F statistic calculated in analysis of variance.

**Figure 7:**
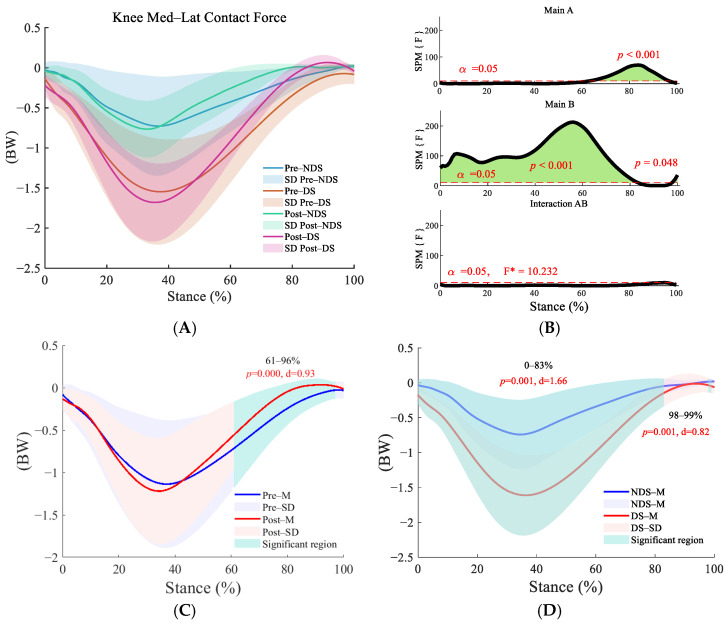
Analysis of medial–lateral contact forces at the knee joint. (**A**): Mean ± SD curves for dominant and non-dominant sides before and after running; (**B**): analysis of main effects of time and side, and their interaction. Black solid lines represent SPM[F] statistics, red dashed lines denote significance thresholds, and green shaded areas indicate regions of statistical significance; (**C**): simple effects analysis before and after running; (**D**): simple effects analysis for dominant and non-dominant sides. M denotes the mean, and SD denotes the standard deviation. “SPM [F]” is clarified as “statistical parametric mapping F-statistic curve.” F* is the F statistic calculated in analysis of variance.

In both the superior–inferior (Figure 6B) and medial–lateral (Figure 7B) directions, the interaction effect was nonsignificant, but the main effects of time and side were significant (*p* < 0.01). Post-run contact forces significantly increased compared to pre-run during the initial support phase (superior–inferior: 15–39%) and push-off phase (superior–inferior: 65–89%, Figure 6C; medial–lateral: 61–96%, Figure 7C) (*p* < 0.001). The dominant side exhibited significantly higher contact forces than the non-dominant side during the push-off phase (superior–inferior: 40–93%, Figure 6D; medial–lateral: 0–83%, 98–99%, Figure 7D); *p* < 0.001), indicating consistent lateral differences.

### 3.3. Bilateral Hip Contact Force Before and After Running

**Figure 8:**
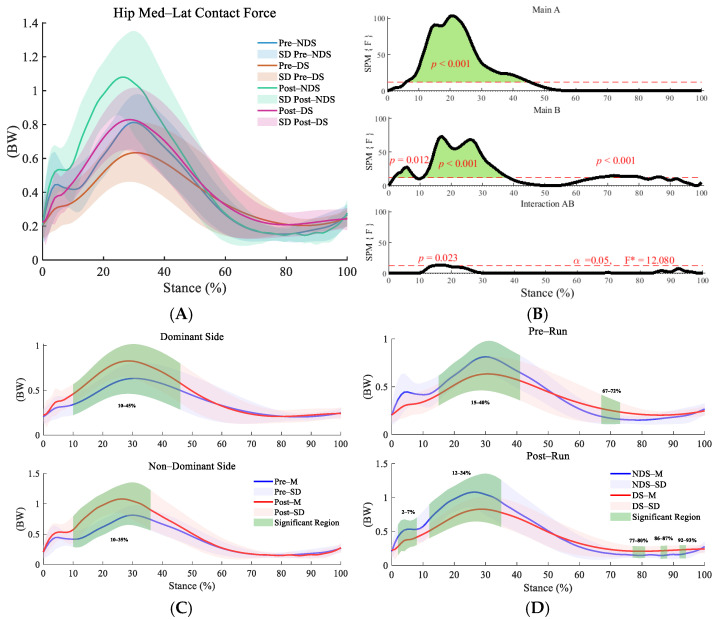
Analysis of medial–lateral contact forces at the hip joint. (**A**): Mean ± SD curves for dominant and non-dominant sides before and after running; (**B**): analysis of main effects of time and side, and their interaction. Black solid lines represent SPM [F] statistics, red dashed lines denote significance thresholds, and green shaded areas indicate regions of statistical significance; (**C**): simple effects analysis for dominant and non-dominant sides; (**D**): simple effects analysis before and after running. M denotes the mean, and SD denotes the standard deviation. “SPM [F]” is clarified as “statistical parametric mapping F-statistic curve.” F* is the F statistic calculated in analysis of variance.

In the medial–lateral direction, the interaction effect between time and side was significant (F = 12.08, *p* = 0.023, η^2^ = 0.139, Figure 8B). Post hoc tests revealed that hip contact forces on both the dominant and non-dominant sides significantly increased during the initial stance phase (dominant side: 10–45%; non-dominant side: 10–35%, Figure 8C) (*p* < 0.01). Intrinsic differences between sides existed during the 15–40% and 67–72% phases before running (*p* < 0.001). Post-running differences further emerged during the initial stance phase (2–7%, 12–34%) and push-off phase (77–80%, 86–87%, 92–93%, Figure 8D).

**Figure 9:**
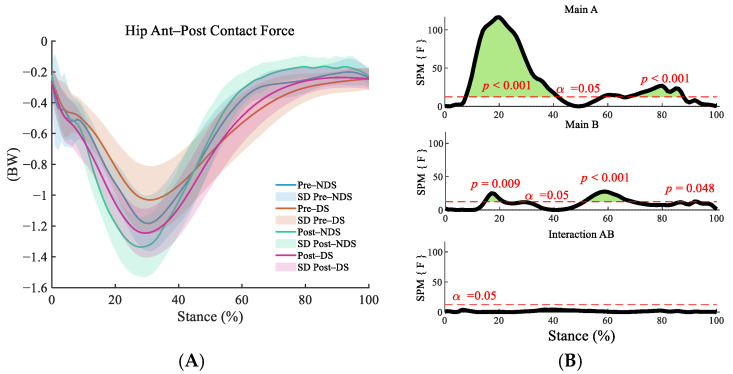

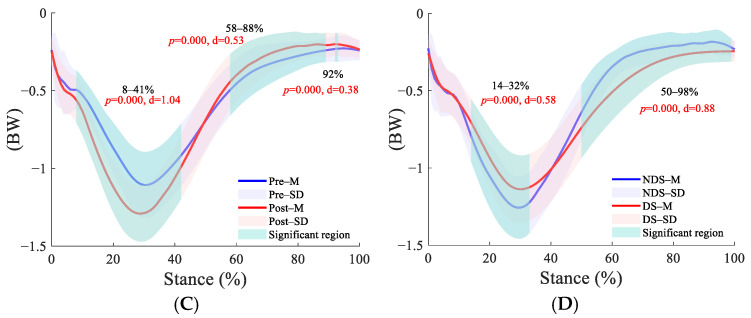
Analysis of superior–inferior contact forces at the hip joint. (**A**): Mean ± SD curves for dominant and non-dominant sides before and after running; (**B**): analysis of main effects of time and side, and their interaction. Black solid lines represent SPM [F] statistics, red dashed lines denote significance thresholds, and green shaded areas indicate regions of statistical significance; (**C**): simple effects analysis before and after running; (**D**): simple effects analysis for dominant and non-dominant sides. M denotes the mean, and SD denotes the standard deviation. “SPM [F]” is clarified as “statistical parametric mapping F-statistic curve”.

**Figure 10:**
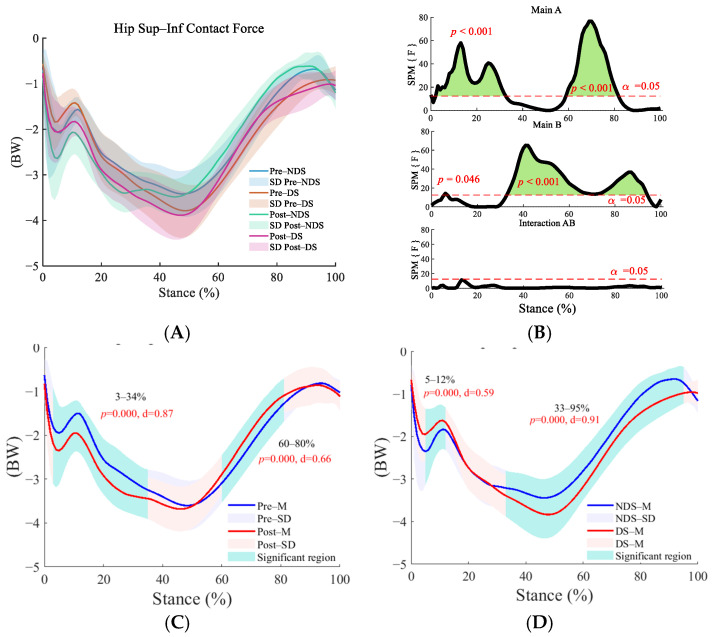
Analysis of anterior–posterior contact forces at the hip joint. (**A**): Mean ± SD curves for dominant and non-dominant sides before and after running; (**B**): analysis of main effects of time and side, and their interaction. Black solid lines represent SPM [F] statistics, red dashed lines denote significance thresholds, and green shaded areas indicate regions of statistical significance; (**C**): simple effects analysis before and after running; (**D**): simple effects analysis for dominant and non-dominant sides. M denotes the mean, and SD denotes the standard deviation. “SPM [F]” is clarified as “statistical parametric mapping F-statistic curve”.

In both the anterior–posterior (Figure 9B) and superior–inferior (Figure 10B) directions, interaction effects were insignificant, but main effects of time and side were significant (*p* < 0.01). Superior–inferior post-run contact forces significantly increased compared to pre-run during early stance (8–41%) and push-off (58–88%, Figure 9C, *p* < 0.001). The dominant side exhibited significantly higher values than the non-dominant side during the initial stance phase (anterior–posterior: 14–32%, Figure 9D; superior–inferior: 5–12%, Figure 10D) and push-off phase (anterior–posterior: 50–98%, Figure 9D; superior–inferior: 33–95%, Figure 10D, *p* < 0.001), indicating consistent lateral differences.

### 3.4. Peak Contact Forces at Bilateral Lower Limb Joints Before and After Running

Table 1 compares peak joint contact forces at three lower limb joints between the dominant and non-dominant sides before and after long-distance running. Analysis reveals an interaction effect between running state and side on lower-limb-joint contact forces. In the anterior–posterior direction, ankle contact forces on the dominant side were significantly greater than those on the non-dominant side both before and after running (*p* < 0.05). Hip contact forces on the dominant side significantly increased after running compared to before (*p* < 0.001). In the superior–inferior direction, contact forces at all three joints significantly increased after running compared to before (*p* < 0.05), with knee contact forces on the dominant side peaking after running. In the medial–lateral direction, contact forces at the dominant knee were significantly greater than those at the non-dominant knee both before and after running (*p* < 0.001), while contact forces at the dominant hip were significantly less than those at the non-dominant hip (*p* < 0.001). Additionally, contact forces on the medial and lateral of the non-dominant hip joint significantly increased after running.

## 4. Discussion

The purpose of this study was to investigate the effects of long-distance running on the joint loads of the dominant and nondominant sides of the lower extremities. The primary findings of this study indicate that contact forces at the hip, knee, and ankle joints of the lower limbs generally increase following long-distance running. Furthermore, asymmetry between the dominant and non-dominant sides significantly worsens during the initial support phase and push-off phase.

The observed increase in multi-joint loading was likely a direct consequence of muscle fatigue and subsequent compensatory strategies. After long-distance running, the fatigue of key lower limb muscles—such as the gluteals, quadriceps, and plantar flexors—impairs their contraction efficiency and rate. To maintain the required propulsion and stability, the neuromuscular system may adopt compensatory activation patterns, leading to elevated muscular forces. Since muscles are primary contributors to joint contact forces, this results in the significant post-run increases we observed across all three joints [24,25]. For instance, the heightened compressive forces at the hip and knee align with the known roles of the gluteus and quadriceps muscles [25], while the increased forces at the ankle, including shear components in the anterior–posterior and medial–lateral directions, are consistent with altered demands on the plantar flexor and tibial anterior [21]. Elevated shear forces are of particular concern as they have been shown to damage deeper regions of cartilage [26,27], and our findings suggest that prolonged exercise may exacerbate mechanical instability at the ankle, a mechanism previously observed in populations with chronic ankle instability [13].

Crucially, the study revealed that fatigue not only increases load but also redistributes it asymmetrically, which may pose a greater injury risk than increased load alone. We found that bilateral asymmetry was aggravated in critical phases of gait, such as the push-off phase for the ankle and hip. This is particularly noteworthy for the asymmetric vertical (compressive) loading at the knee and hip during push-off. Given that compressive forces are directly associated with joint cartilage wear [25] and the development of stress fractures [28], this specific asymmetry indicates a substantially elevated risk for these common running-related injuries. The underlying cause may be fatigue-induced breakdown in motor control [29], leading to a loss of optimal inter-joint coordination [30,31]. This is supported by the “work redistribution” theory, where fatigue causes the proximal joints (knee and hip) to compensate for a fatigued ankle [30], a process that may be inherently asymmetrical due to pre-existing limb dominance [32]. Furthermore, the asymmetry in ankle contact force across all three directions may be linked to changes in foot posture after prolonged exercise. Studies indicate that long-distance runners exhibit an asymmetrical range of motion in foot inversion and eversion, and such asymmetries in foot valgus can severely impact attenuation, thereby increasing the risk of overuse injuries [33]. The fact that the dominant leg generally provides greater propulsive force [32] corroborates our finding of consistently higher joint contact forces on the dominant side.

From a kinetic chain perspective, loading changes at the hip, knee, and ankle are interdependent. During running, these three joints coordinate to produce movement, and an alteration in one joint inevitably influences the loading of the others [34]. The increases and asymmetric shifts in contact force observed in this study are thus likely interactive. In long-distance, cyclic exercise, as muscle fatigue develops, the body produces compensatory movements aimed at maintaining economy. However, these compensations can simultaneously lead to excessive and asymmetrical loading patterns. This creates a maladaptive cycle where intensifying asymmetry exposes the dominant limb to repetitive, excessive loads that accumulate over time [35,36,37]. Although individual loading cycles may not exceed acute injury thresholds, the accumulation of these loads is a key etiological factor in running-related injuries [28,37]. When cumulative loads increase, fundamental gait metrics such as symmetry and joint stability are altered, further elevating injury risk [38,39].

In conclusion, long-distance running induces a detrimental cycle of increased joint loading and exacerbated bilateral asymmetry, driven by muscle fatigue and compromised motor control. Therefore, identifying these dynamic changes is crucial. Our findings provide a biomechanical rationale for runners and coaches to move beyond viewing fatigue only as a metabolic state but also as a significant neuromusculoskeletal risk factor. Monitoring fatigue and incorporating training interventions aimed at improving bilateral balance, inter-joint coordination, and fatigue resistance are essential steps to mitigate the identified injury risks.

## 5. Conclusions

Long-distance running not only increases multi-joint loading on the lower limbs but also exacerbates asymmetry between the dominant and non-dominant sides during the initial support phase and push-off phase. This redistribution of load and bilateral control imbalance may further elevate injury risk. For practical applications, these findings suggest that coaches and clinicians should (1) incorporate bilateral movement assessments into routine evaluations to identify asymmetry; (2) design targeted training programs that include unilateral strengthening and stability exercises, particularly for the non-dominant limb; and (3) consider individual asymmetry profiles when prescribing training loads. The observed asymmetry patterns provide a biomechanical rationale for such interventions, although their efficacy in injury prevention warrants confirmation in future longitudinal studies.

## Figures and Tables

**Table 1:** Peak joint contact forces at the three lower limb joints on the dominant and non-dominant sides before and after long-distance running, supplementing the continuous SPM1d analysis.

	Variables	Pre-Long-Distance Running		Post-Long-Distance Running		Time × Side Interaction
	Joint Contact Force	Non-Dominant	Dominant	Non-Dominant	Dominant	*p*-Value (η^2^)
Ankle	Anterior–Posterior	0.49 ± 0.64	0.83 ± 0.92 #	0.64 ± 0.92	1.16 ± 1.14 #	0.119 (0.12)
	Superior–Inferior	3.05 ± 1.28	3.22 ± 1.47	3.94 ± 1.79 *	4.23 ± 1.87 *	0.086 (0.15)
	Medial–Lateral	0.22 ± 0.09	0.20 ± 0.05	0.26 ± 0.09 *	0.21 ± 0.06	0.137 (0.11)
Knee	Anterior–Posterior	0.44 ± 0.12	0.43 ± 0.16	0.45 ± 0.12	0.54 ± 0.17	0.808 (0.00)
	Superior–Inferior	8.02 ± 1.90	7.93 ± 1.58	8.35 ± 1.31 *	8.81 ± 1.34 *	0.819 (0.00)
	Medial–Lateral	0.83 ± 0.47	1.56 ± 0.66 #	0.78 ± 0.35	1.69 ± 0.48 #	0.000 (0.66) †
Hip	Anterior–Posterior	1.23 ± 0.17	1.06 ± 0.21 #	1.38 ± 0.18 *	1.26 ± 0.15 *#	0.016 (0.27) †
	Superior–Inferior	3.59 ± 0.56	3.84 ± 0.56 #	3.75 ± 0.33 *	4.01 ± 0.49 *#	0.038 (0.21) †
	Medial–Lateral	0.84 ± 0.16	0.66 ± 0.18 #	1.14 ± 0.29 *	0.85 ± 0.19 *#	0.000 (0.69) †

Notes: Outcome variables are shown as the means ± SD(BW); † significant time × side interaction effect (*p* < 0.05); * indicates comparisons between running before and after on the same side (*p* < 0.05); # indicates comparisons between the dominant and non-dominant sides at the same time point (*p* < 0.05).

## Data Availability

The data presented in this study are available on request from the corresponding author.
